# The Genome of *Bifidobacterium longum* subsp. *infantis* YLGB-1496 Provides Insights into Its Carbohydrate Utilization and Genetic Stability

**DOI:** 10.3390/genes15040466

**Published:** 2024-04-08

**Authors:** Xiaoxia Li, Jianjun Yang, Shaoqi Shi, Hanglian Lan, Wen Zhao, Weilian Hung, Jian He, Ran Wang

**Affiliations:** 1 Research Center for Probiotics, Department of Nutrition and Health, China Agricultural University, Beijing 100190, China; lixiaoxia0154@163.com (X.L.); 18811626398@163.com (J.Y.); ssqshishaoqi@163.com (S.S.); zhaowen@yili.com (W.Z.); 2 National Center of Technology Innovation for Dairy, Hohhot 010110, China; lanhanglian@yili.com (H.L.); hungweilian@yili.com (W.H.); hejian@yili.com (J.H.)

**Keywords:** *Bifidobacterium longum* subsp. *infantis*, glycoside hydrolases, continuous subculture, genetic stability

## Abstract

*Bifidobacterium longum* subsp. *infantis* YLGB-1496 (YLGB-1496) is a probiotic strain isolated from human breast milk. The application of YLGB-1496 is influenced by carbohydrate utilization and genetic stability. This study used genome sequencing and morphology during continuous subculture to determine the carbohydrate utilization characteristics and genetic stability of YLGB-1496. The complete genome sequence of YLGB-1496 consists of 2,758,242 base pairs, 2442 coding sequences, and a GC content of 59.87%. A comparison of carbohydrate transport and metabolism genes of *Bifidobacterium longum* subsp. *infantis* (*B. infantis*) showed that YLGB-1496 was rich in glycosyl hydrolase 13, 20, 25, and 109 gene families. During continuous subculture, the growth characteristics and fermentation activity of the strain were highly stable. The bacterial cell surface and edges of the 1000th-generation strains were progressively smoother and well-defined, with no perforations or breaks in the cell wall. There were 20 SNP loci at the 1000th generation, fulfilling the requirement of belonging to the same strain. The presence of genes associated with cell adhesion and the absence of resistance genes supported the probiotic characteristics of the strain. The data obtained in this study provide insights into broad-spectrum carbohydrate utilization, genomic stability, and probiotic properties of YLGB-1496, which provide theoretical support to promote the use of YLGB-1496.

## 1. Introduction

Probiotic development and consumption have increased globally in recent decades because of awareness of the beneficial effects their use has on promoting gut and overall health [1]. Probiotics are living bacteria that, when consumed in sufficient quantities, provide health benefits to the host [2]. The *Lactobacillus* group and *Bifidobacterium* genus are probiotic microorganisms that are most often utilized [3]. The *Bifidobacteria* genus is Gram-positive, nonmotile, non-sporulating, heterofermentative, obligate anaerobes that are commonly found in the gastrointestinal tract, breast milk, and vagina [4,5]. The genus contains approximately 50 species, of which only 10 are found in humans. Some strains of the *Bifidobacterium* genus are thought to benefit the host by stimulating and modulating both innate and adaptive host immune responses, reducing the inflammatory response, and degrading diet-derived carbohydrates [6].

As an important member of the bifidobacteria, *Bifidobacterium longum* (*B. longum*)species are classified into four subspecies: *B. longum* subsp. *longum*, *B. longum* subsp. *suis*, *B. longum* subsp. *suillum*, and *B. longum* subsp. *infantis* (*B. infantis*) [7]. The fact that members of *B. infantis* have been found in breast milk, infant guts, and breastfed newborn feces suggests that they have a good impact on human health [4,5]. It has been shown that five gene clusters in *B. infantis* may efficiently employ certain human milk oligosaccharides (HMOs) for intracellular metabolism and transport [8,9]. In addition, *B. infantis* has a large number of glycosyl hydrolases (GH), which are hydrolases that metabolize oligosaccharides originating from plants or milk, including indigestible ones like galactooligosaccharides and fructooligosaccharides [10]. *B. infantis* can effectively collect preferred carbon sources in the competitive ecosystem of infants’ guts thanks to these transporter-dependent intracellular consumption methods, demonstrating the irreplaceable probiotic role of this strain in the host–microbe co-evolution of infants [11,12].

*B. infantis* R0033 [13], *B. infantis* ATCC15697 [9], *B. infantis* EVC001 [14], *B. infantis* CECT7210 [15], *B. infantis* 35624 [16], and *B. infantis* BI-G201 [17] are mainly used in infant-related foods, such as infant supplemented formula, solid beverages, and probiotic dietary supplements. It is crucial to employ *B. infantis* in milk-borne molecules that lack a nutritive value to the neonate to utilize HMOs, and it is clear that some *Bifidobacterium animalis* (*B. animalis*) strains lack this capacity [13]. For a commercial strain, the ability to maintain probiotic properties and genetic stability is the primary condition and criterion for industrialization. The sequencing of *B. infantis* is essential since it is an attractive candidate for use in infant probiotics [18,19]. Whether the strain can be transferred from laboratory to industrial production depends greatly on the strain’s continuous-subculture stability [20].

This study aimed to clarify the carbohydrate utilization and genetic stability of *Bifidobacterium longum* subsp. *infantis* YLGB-1496 (YLGB-1496) by analyzing its genome and consecutive subcultures; we isolated YLGB-1496 with prospective uses for its cell-free culture supernatant, which exhibited strong oxidation resistance and enhanced skin barrier function [2]. We sequenced the whole genome of YLGB-1496 (China Center for Type Culture Collection: No. M2011122) and compared it with the genomes of other *B. infantis* strains to understand its carbohydrate utilization capacity and probiotic characteristics. Using next-generation technologies, we performed deep sequencing on wild YLGB-1496 and consecutive subcultures to look for low-frequency variants and to evaluate genome stability.

## 2. Materials and Methods

### 2.1. Bacterial Growth Conditions

YLGB-1496 was initially isolated from human breast milk by our team, and the isolate was stored in cryoprotectant (12% (*w*/*v*) skim milk containing 10% glycerol) at −80 °C. YLGB-1496 was propagated twice in de Man, Rogosa, and Sharpe (MRS) broth (Oxoid Ltd., Beijing, China) at 37 °C overnight in an anaerobic incubator.

### 2.2. Identification of Novel YLGB-1496

The strain was incubated in an anaerobic atmosphere (2.99% H_2_, 17.01% CO_2_, and 80% N_2_) for 16 h at 37 °C in a chamber with MRS supplemented with 0.05% (*w*/*v*) L-cysteine hydrochloride [2020]. Subsequently, the cells were harvested via centrifugation at 10,000× *g* for 5 min, and the obtained cell pellet was washed with TES buffer (50 mM Tris-Cl, 30 mM EDTA, 25% sucrose, pH 8.0) [2121]. The manufacturer’s instructions were then followed to extract DNA using the E.Z.N.A. Bacterial DNA Kit (Omega Bio-tek, Norcross, GA, USA).

Taxonomic identification was confirmed by sequencing of PCR-amplified 16S rRNA using the universal primers Blong1 5′-TCCCAGTTGATCGCATGGTC-3′ and Blong 5′-GGGAAGCCGTATCTCTACGA-3′ [21]. *B. longum* species-specific strain was specifically identified with 31 housekeeping loci (Table S1) [22,23]. Obtained sequences were compared with reference sequences in GenBank using the Basic Local Alignment Search Tool (BLAST).

### 2.3. Carb Trials

The fermentation substrate concerning the mono/oligosaccharide range was determined with the API 20A assay (Biomérieux, Marcy l’Etoile, France) [24]. Active strain cultures were washed twice with sterile physiological saline (0.9% *w*/*v*, NaCl), and pellets were resuspended in API 20A medium (BioMérieux, SA, Marcy l’Etoile, France). Homogenized cell suspensions were transferred into 50 wells of the API 50 CH strips using sterile Pasteur pipettes. All wells were overlaid with sterile mineral oil (Sigma, St. Louis, MO, USA) to effect anaerobiosis. Strips were moistened and incubated at 37 °C for 24–48 h.

Further validation of specific sugar sources. The strain was inoculated at 1 × 10^7^ CFU/mL in an anaerobic atmosphere in a chamber in MRS containing 0.5% of a carbohydrate carbon source supplemented with 0.05% (*w*/*v*) L-cysteine hydrochloride. All incubations were carried out at 37 °C and in quadruplicate. We considered that the strain grows on the tested sugar when the turbidity at 600 nm reaches more than 0.5. The blank is represented by MRS without bacteria.

### 2.4. Continuous Subculture of YLGB-1496

The strain was injected in MRS broth at 2% (*v*/*v*) and continuously incubated for up to 1000th generation. Twelve hours was defined as one continuous cycle of culture. According to the strain’s growth curve, the number of bacteria increases approximately 50-fold at 2% inoculum, with log_2_ 50 ≈ 5.64 generations of growth (i.e., the number of times the bacteria divided) in each cycle [25]. The absorbance of the culture solution at 600 nm was measured every 200 generations using a UV-2800A spectrophotometer (Unico Instrument Co., Ltd., Beijing, China). At the same time, colonies were observed for morphology, size, and color. All experiments were performed in three biological replicates.

### 2.5. Viable Microbial Counts

The count of strain was determined by counting colonies on MRS agar supplemented with 0.05% (*w*/*v*) L-cysteine hydrochloride in an anaerobic atmosphere in a chamber at 37 °C, as described previously [21,23]. The number of viable cells per gram was counted and expressed as lg CFU/mL.

### 2.6. Scanning Electron Microscopy (SEM) Analysis of YLGB-1496

The strain-normal culture or continuous subculture was harvested via centrifugation at 1000× *g* for 10 min. After washing twice with 10 mmol/L phosphate-buffered saline (PBS, 137 mM NaCl, 2.7 mM KCl, 10 mM Na_2_HPO_4_, 2 mM KH_2_PO_4_, pH 7.4), cell pellets were resuspended to 1 × 10^5^ CFU. Bacterial pellets were then fixed overnight in 500 mL of 2.5% (*v*/*v*) glutaraldehyde in PBS at 4 °C. Following that, the bacteria were rinsed twice with PBS and dehydrated for 15 min in a graded ethanol series (50%, 70%, 90%, and 100%). The samples were then transferred for 20 min to a mixture (1.1, *v*/*v*) of ethanol, tertiary butanol, and pure tertiary butanol. After lyophilization and gold coating, the specimens were observed using SEM (Hitachi SU70, Tokyo, Japan).

### 2.7. Determination of the Fermentation Viability

The milk fermentation medium was reconstituted skim milk powder (10% (*w*/*v*); Fonterra^TM^, Auckland, New Zealand) with glucose (2% (*w*/*v*)). The medium was sterilized by heating for 15 min at 115 °C and inoculated with 1 × 10^7^ CFU/mL of strain, followed by incubation at 37 °C for 24 h. The milk fermentation viability of the strain was determined according to the China National Standard GB 5009.239-2016, as described previously [26]. One viability unit (U) was calculated as 1 μmol of lactic acid per 1 × 10^7^ CFU of bacterial cells fermenting 1 mL of milk under the above conditions.

### 2.8. YLGB-1496 Genome Sequencing and Assemblies

The genome sequence analysis of YLGB-1496 was carried out using the Illumina NovaSeq6000 sequencing platform (MajorBio Co., Shanghai, China). In brief, DNA samples were sheared into 400–500 base pairs (bp) fragments using a Covaris M220 Focused Acoustic Shearer, following the manufacturer’s protocol. Illumina sequencing libraries were prepared from the sheared fragments using the NEXTflex™ Rapid DNA-Seq Kit. The prepared libraries were used for paired-end Illumina sequencing (2 × 150 bp). Raw reads obtained after sequencing were filtered using fastp software [27] followed by assembly with SOPA de novo [28]. Glimmer [29] was used for CDS prediction, tRNA-scan-SE [30] was used for tRNA prediction, and Barrnap (https://github.com/tseemann/barrnap/, accessed on 1 January 2024) was used for rRNA prediction. The predicted CDSs were annotated from NR, Swiss-Prot (https://web.expasy.org/docs/swiss-prot_guideline.html, accessed on 1 January 2024), Pfam (http://pfam.xfam.org/, accessed on 1 January 2024), GO (http://current.geneontology.org/ontology/index.html, accessed on 1 January 2024), COG (http://eggnogdb.embl.de/#/app/home, accessed on 1 January 2024), and KEGG databases (http://www.genome.jp/kegg/, accessed on 1 January 2024) using sequence alignment tools such as BLASTP (http://ftp.ncbi.nlm.nih.gov/blast/executables/blast+/2.3.0/, accessed on 1 January 2024), Diamond (https://github.com/bbuchfink/diamond, accessed on 1 January 2024), and HMMER (http://www.hmmer.org/, accessed on 1 January 2024).

### 2.9. Statistical Analyses

The data were log2 transformed before analysis. Cluster analysis of YLGB-1496 was performed using IBM SPSS statistics. Each experiment was performed in triplicate. Statistical analyses were performed using SPSS13.0 software (SPSS Inc, Chicago, IL, USA). All results are expressed as a mean ± standard deviation (SD). Significant differences between the sample means were determined at *p* < 0.05 using a Tukey’s test.

### 2.10. Sequence Analysis, Software, and Databases

Similarity searches of all the predicted proteins were performed against the non-redundant database/NCBI and Swiss-Prot/EMBL and the toxin and virulence factor database MvirDB [31] using BLASTP (E-value ≤ 10^−10^, identity ≥ 30%, coverage ≥ 30%). Functional classification of protein-coding genes was performed using NCBI clusters of orthologous groups (COGs) using BLASTP (E-value ≤ 10^−10^, identity ≥ 30%, coverage ≥ 30%). tRNA-scan-SE was used for tRNA prediction, Barrnap was used for rRNA prediction, and the analysis of RNA modifications was conducted by second- and third-generation deep sequencing [32]. Sequence similarity was detected with BLAST, and multiple sequence alignments were performed using Clustal [33]. Putative sugar and amino acid metabolic pathways were predicted using KEGG (http://www.genome.jp, accessed on 1 January 2024). Carbohydrate-active enzymes were identified using the CAZy database (http://www.cazy.org, accessed on 1 January 2024) [34]. The prediction of secreted proteins was performed using signalP (http://www.cbs.dtu.dk/services/SignalP, accessed on 1 January 2024). TMHMM (http://www.cbs.dtu.dk/services/TMHMM/, accessed on 1 January 2024) was used to search for transmembrane helix structures. A comparison of transporter-protein-related genes in the samples was carried out via the TCDB database (http://www.tcdb.org/, accessed on 1 January 2024). PHAge (PHAST) (http://phast.wishartlab.com, accessed on 1 January 2024) was used for the detection of prophage sequences in bacterial genomes or plasmids [35]. BWA (http://bio-bwa.sourceforge.net/, accessed on 1 January 2024) was used to compare the reads obtained from sequencing with the reference genome sequence for sequencing depth and coverage statistics [36]. Snippy4.6.0 (https://github.com/tseemann/snippy, accessed on 1 January 2024) was used to assess single-nucleotide polymorphisms (SNPs) and other information detection. SnpEff (http://snpeff.sourceforge.net/SnpEff.html, accessed on 1 January 2024) was used for variant locus annotation [37]. CARD (http://arpcard.Mcmaster.ca, accessed on 1 January 2024, Version 1.1.3) was utilized for the prediction of drug-resistant genes [38]. Average nucleotide concordance analysis was performed using the Majorbio Cloud Platform (https://cloud.majorbio.com/page/tools/, accessed on 1 January 2024) [39].

## 3. Results

### 3.1. Genome Sequences of YLGB-1496

**Figure 1:**
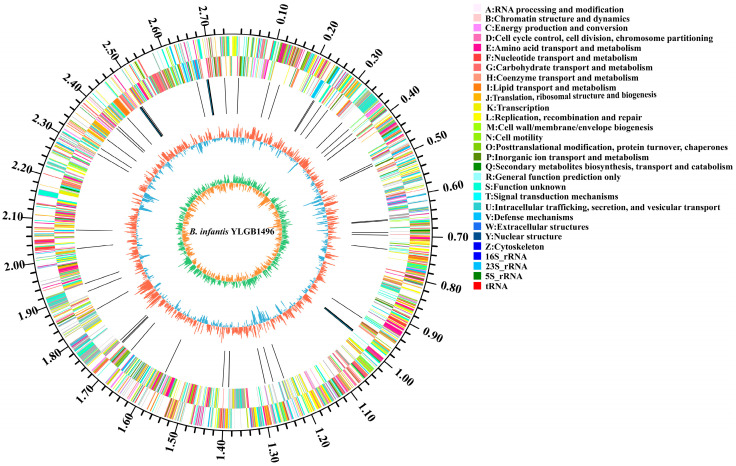
The figure shows a circular map of *Bifidobacterium longum* subsp. *infantis* YLGB-1496 (YLGB-1496). From outside to inside, the outermost circle of the circle diagram was the identification of genome size. The second and third circles were the CDS on the positive and negative strands, with various colors indicating different COG functional classes. The fourth circle was the rRNAs and tRNAs. The fifth circle was the GC content, and the outward red portion indicates that the GC content of the region was higher than the average GC content of the whole genome, while the inward blue part indicates that the GC content of the region was lower than the genome-wide average GC content, and higher peaks indicate a larger difference from the average GC content. The innermost circle was the GC-Skew value, and the specific algorithm is G-C/G+C, which could help to determine the leading and trailing strand; generally, the leading strand GC skew > 0 and the lagging strand GC skew < 0, and also assist in determining the replication start point (cumulative offset minimum) and the end point (cumulative offset maximum), which was especially important for the circular genome.

According to the findings of this study, the genome of YLGB-1496 consists of single circular chromosomes of 2,758,242 bp and has no plasmid (Figure 1). The GC content was 59.87%, which was comparable to that of other strains of *B. infantis* [4,9]. The genome contains 2442 predicted coding sequences (CDSs) and 64 structural RNAs (6 rRNA and 58 tRNA). No active phages were found by PHAge in the YLGB-1496 genomic sequences (Table 1).

### 3.2. Phylogenetic Analysis of YLGB-1496

**Figure 2:**
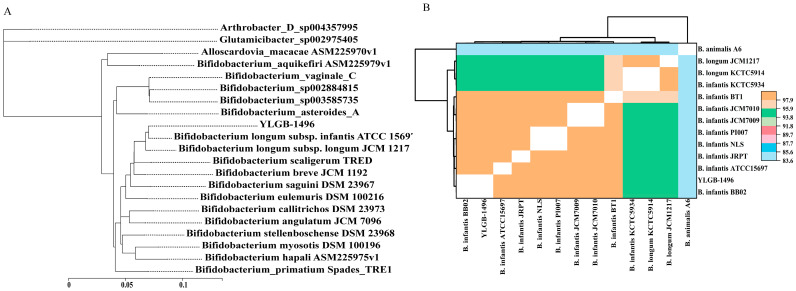
(**A**) The figure shows the phylogenomic analysis using whole-genome sequences of closely related strains of YLGB-1496. The 31 housekeeping genes (*dnaG*, *frr*, *infC*, *nusA*, *pgk*, *pyrG*, *rplA*, *rplB*, *rplC*, *rplD*, *rplE*, *rplF*, *rplK*, *rplL*, *rplM*, *rplN*, *rplP*, *rplS*, *rplT*, *rpmA*, *rpoB*, *rpsB*, *rpsC*, *rpsE*, *rpsI*, *rpsJ*, *rpsK*, *rpsM*, *rpsS*, *smpB*, and *tsf*) were selected as the 19 closest strains at the species level, and the neighbor-joining (NJ) method was used to construct a phylogenetic tree; (**B**) heatmaps of ANI values of the 13 genomes.

The genome-wide comparative analysis of selected strains from different *Bifidobacterium* species strains represents phylogenomic data (Figure 2A). The phylogenomic analysis shows that within the *Bifidobacterium* genus, YLGB-1496 is most closely related to *B. infantis* ATCC 15697, which was isolated from an infant’s feces [40]. It indicated that YLGB-1496 had some similarities to strains reported in the literature. To detect strain boundaries, pairwise genome comparisons were performed using average nucleotide identity (ANI) metrics (Figure 2B); the ANI values of all strains were between 83.56% and 99.90%, and it evidenced the presence of three distinct clades: one represented by *B. animalis* A6, another represented by *B. infantis* KCTC5934 and two *B. longum* JCM1217 and KCTC5934, and the third clades comprising the remaining nine *B. infantis* genomes, including YLGB-1496 (Figure 2B). However, a more in-depth study of the genomes of such isolates is required to better discover the genetic differences that lead to carbohydrate utilization characteristics.

### 3.3. Genetic and Phenotypic Data for Carbohydrate Metabolism

**Figure 3:**
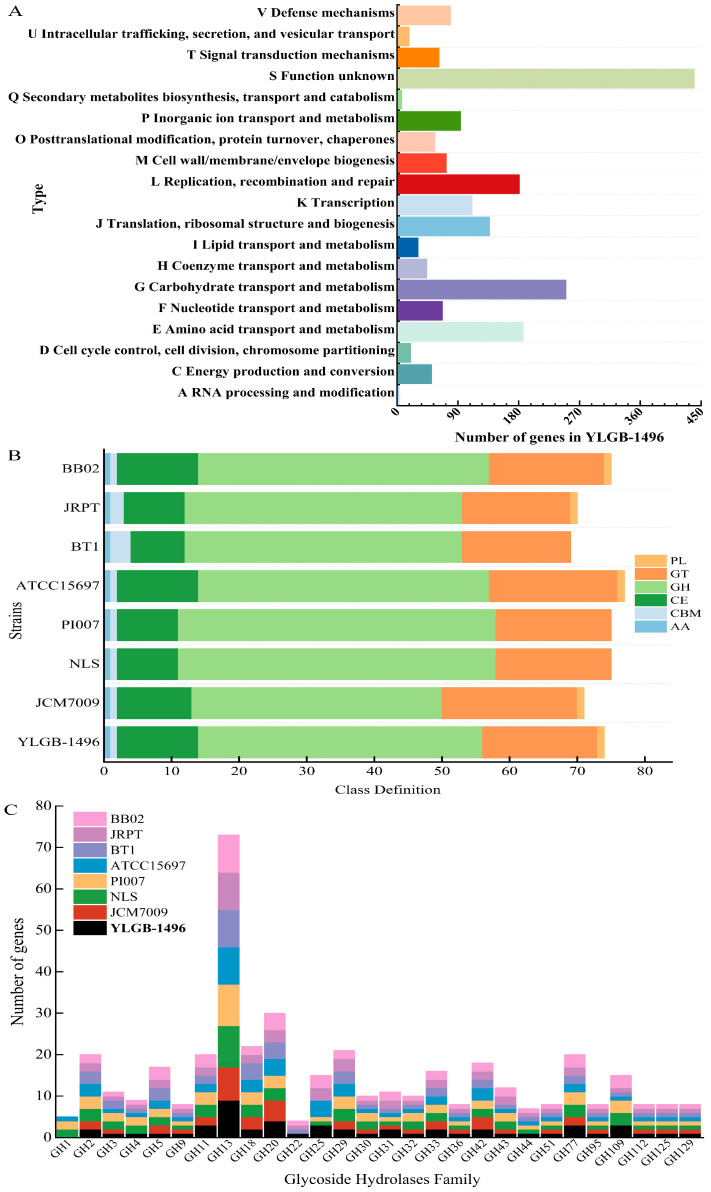
The figure shows the genetic data for carbohydrate metabolism. (**A**) COG function classification of genes for YLGB-1496. The genome was mapped to the bacterial COGs and KEGG database to evaluate the main functional COG categories related to the genome. (**B**) Carbohydrate-active enzymes classification of eight *B. infantis* strains. The genes of the eight *B. infantis* strains were comparatively analyzed using Diamond and hmmscan software after annotation through the CAZy Database (http://www.cazy.org/, accessed on 1 January 2024) (E-value ≤ 10^−5^). PL: Polysaccharide Lyases; GT: Glycosyl Transferases; GH: Glycoside Hydrolases; CE: Carbohydrate Esterases; CBM: Carbohydrate-Binding Modules; AA: Auxiliary Activities. (**C**) Glycoside hydrolases family of eight *B. infantis* strains.

The protein sequences of the YLGB-1496 genome were subjected to an orthology prediction using OrthoMCL, with thresholds of E-Value: 1e-5, percent identity cutoff: 0, and Markov inflation index: 1.5. Compared to the other seven *B. infantis* strains, YLGB-1496 had the highest number of genes clustered to COG (Table 1). The 1896 genes in the YLGB-1496 genome were mapped to the bacterial COG and KEGG databases [41,42] to assess the main functional COG categories associated with the genome (Table S1). The major fraction of ortho groups (23.02%) were grouped under the category of “unknown function” (S) (Figure 3A), this assignment indicating the need for additional advances in functional gene prediction. However, 13.08% of the OGs were classified as “carbohydrate transport and metabolism” (G), which showed that the ability to utilize a wide range of carbohydrates is an important attribute of the organism (Figure 3A). Further analysis of the G category genes (209 genes) through the Cazy database revealed that of the 74 genes mapped to the Cazy database, GHs (56.76%) were the most abundant, followed by glycosyl transferases (22.97%) and carbohydrate esterases (16.22%) (Figure 3B). The GH13 family was the most abundant glycoside hydrolase in YLGB-1496, followed by G11, G20, and G25. Furthermore, the GH36, GH42, GH51, and GH77 families were discovered to be specifically present in *Bifidobacterium* (Figure 3C).

**Figure 4:**
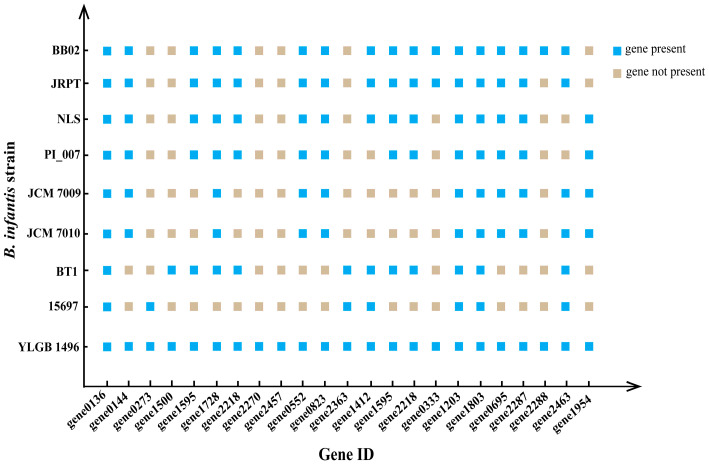
The figure shows the glycoside hydrolase-specific genes of YLGB-1496.

We compared the glycoside hydrolase genes in YLGB-1496 and seven *B. infantis* strains (Figure 4, Table S2). The largest number of genes from the GH13 family were found in YLGB-1496, of which α-amylase, α-glucosidase, and α-1,4-glucan:maltose -1-phosphate maltosyltransferase are typical representatives; within the GH20 family, none of the six *B. infantis* strains has a β-hexosaminidase; LysM peptidoglycan-binding-domain-containing protein in the GH25 family involved in the degradation of bovine lactose macropeptides [43] was also found in *B. infantis* GRPT and *B. infantis* BB02. Nine strains included the GH36 and GH42 families of genes, but none of the seven *B. infantis* strains included inositol 2-dehydrogenase (Figure 4, Table S2).

**Figure 5:**
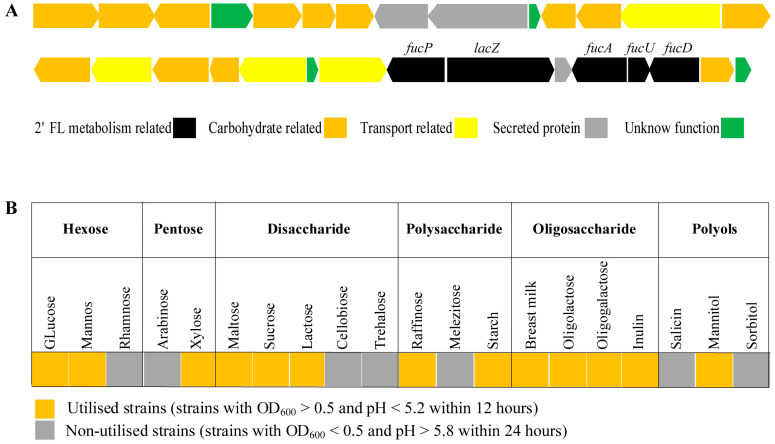
The figure shows the 2′-FL metabolism-related genes and 20 carbohydrate’s utilization of YLGB-1496. (**A**) Annotated contiguous genes related to 2′-FL utilization in YLGB-1496. The cluster containing a *fucP* (fucose permease), *lacZ* (β-galactosidase), *fucA* (α-L-fucosidase), *fucU* (L-fucose mutarotase), *fucD* gene (gene2350) and *fucO* gene (lactaldehyde reductase) [40]. (**B**) YLGB-1496 metabolic capacity for 20 carbohydrates. It is considered that the strain grows on the tested sugar when the turbidity at OD_600_ reaches more than 0.5.

We further analyzed carbohydrate metabolism pathways and found that eight *B. infantis* strains shared the same Leloir and fermentative pathway genes necessary to break down glucose and galactose through the bifid shunt (Table S3). It is also worth noting that a gene cluster was identified in YLGB-1496 containing a *fucP* (gene2316), *lacZ* (gene2344), *fucA* (gene2346), *fucU* (gene2347), and *fucD* gene (gene2350) (Figure 5A). Fucose-utilizing genes are conserved in all tested *B. infantis* genomes, all having the necessary genes to produce 1,2-propanediol (1,2-PD) (Table S4). The main products of both 2′-fucosyllactose (2′-FL) and lactose fermentation can be predicted as acetate, formate, pyruvate, lactate, and 1,2-PD in 2′-FL fermentation. We examined the ability of YLGB-1496 to metabolize 20 carbohydrate substrates verified by a single sugar source. In addition to the basic hexoses and pentoses, the diversity of glycoside hydrolases allows them to utilize a wide range of oligosaccharides, such as breast milk, oligolactose, or oligogalactose (Figure 5B).

### 3.4. Morphology during YLGB-1496 Continuous Subculture

**Figure 6:**
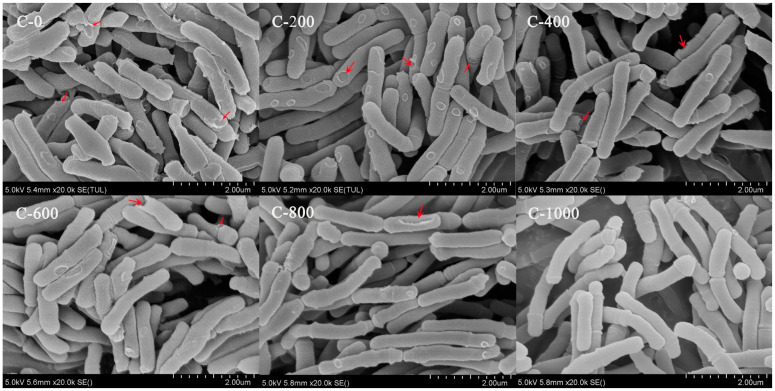
The figure shows the morphology of YLGB-1496 in the continuous subculture. The samples were prepared using ethanol gradient drying, and the surface morphology of the strain was observed using SEM at a magnification of 5000× in the 2 μm range. Red arrows indicate cell wall perforation or fracture.

In consecutive subcultures of the 0th, 200th, 400th, 600th, 800th, and 1000th generations, the colonies of the strain had consistent morphology and were white or yellowish and circular. The colonies were 1–3 mm in diameter, with smooth, raised surfaces and tidy borders (Figure S1A). The morphology was curved or rod-shaped structures that were irregularly arranged (Figure S1B). The colony and morphology of the strain showed the characteristic morphology of *Bifidobacterium* and did not vary significantly across 1000 generations. The microscopic morphology of the strain during successive subcultures was observed through the use of SEM (Figure 6). The results showed that the wild strain had a typical rod-like shape with regular arrangement, as well as partial cell wall perforation and fracture. There was no flagellum or pod membrane on the surface of the bacterium (Figure 6C-0). As the continuous subculture progressed to 1000 generations, the surface and edges of the bacterium gradually became smooth and clear, and the number of perforated and fractured cell walls gradually decreased (Figure 6C-200, 400, 600, and 800), indicating that the morphology of the strain gradually entered a stable state during the continuous subculture up to 1000th generation.

### 3.5. Growth Characteristics during YLGB-1496 Continuous Subculture

The growth characteristics of the strain in continuous subculture for 1000 generations are shown in Table 2. The OD_600_, pH value, and culturable bacteria number of the strain did not change, and the fermentative activity remained largely unchanged at around 60.17 U (*p* > 0.05) in the continuous subculture, indicating that the strain’s overall stability was good and that continuous subculture did not affect the strain’s growth or fermentation.

### 3.6. Stability of Genetic Information

Consecutive subcultures of strain were carried out to compare gene single-nucleotide polymorphism (SNP) analysis via deep sequencing (approx. 1000-fold data coverage). YLGB-1496 had 14 SNP loci at 200 and 400 generations, 18 at 600 and 800 generations, and 20 at 1000 generations, along with two insertional mutations and one deletion (Table 3). The findings demonstrated that the genetic makeup of the strain remained constant throughout 1000 successive generations and satisfied the requirements for belonging to the same strain [44].

### 3.7. Genetic Data for Extracellular Proteins Involved in Adhesion

YLGB-1496 still had its unique adhesion properties. Compared to the nearest related *B. infantis* ATCC 15697, twelve genes, namely, the six flp pilus and four sortase genes, as well as a gene for one tight-adherence protein and the outer-membrane-specific lipoprotein, were identified in YLGB-1496 as being responsible for the probiotic properties of cell adhesion (Table S5). The predicted and functionally annotated secretory (Sec) pathway secreted lipoproteins were found in YLGB-1496, and the results are shown in Table 4. The analysis showed that there were seven Sec pathway-secreted proteins, seven of which contained transmembrane helix structures. Seven of the seventeen genes are involved in amino acid transport and metabolism, seven in carbohydrate transport and metabolism, and three in intracellular trafficking, secretion, and vesicular transport (Table 4).

### 3.8. Drug Resistance Genes in YLGB-1496

We analyzed the whole genome of YLGB-1496, and we found no evidence of any genes for drug resistance to any of the antibiotics tested, including aztreonam, cycloserin, polymyxin B, mupirocin, spectinomycin, kanamycin, gentamicin, streptomycin, doxorubicin B, nalidixic acid, barongomycin, neomycin, tetracycline, and erythromycin. The result demonstrates that YLGB-1496 does not pose a risk for antimicrobial resistance or heteroresistance. Because of the possible impact on human health, Bifidobacterium spp. bacteria should not be allowed to have genes for antimicrobial resistance [45,46]. The ability to pose a risk for antimicrobial resistance or heteroresistance must be considered an important parameter for selecting probiotic strains.

## 4. Discussion

ANI values are based on the pairwise alignment of genomic segments; ANI provides more robust results when the pair of genomes compared share a high degree of similarity (ANI > 90%), an ANI of 95% to 96%, which corresponds to 98.65% 16S rRNA gene sequence similarity, is widely accepted as a threshold for species demarcation [47]. According to the ANI values of 98.40–99.90% for YLGB-1496 and *B. infantis* BB02, ATCC15697, JRPT, NLS, PI007, JCM7009, and BT1, YLGB-1496 is a member of the *B. infantis* species, and there is little diversity within it.

Glycoside hydrolases, widely distributed enzymes that catalyze the cleavage of glycosidic bonds in sugar or sugar conjugates, are considered to be catabolic enzymes that play key roles in carbohydrate metabolism. The GH13 family consists of glycoside hydrolases present in humans, in which the main substrate is an amylopectin-like polysaccharide. The widespread presence of this enzyme in the intestinal flora is indicative of the importance of amylopectins to the intestinal flora [48]. The most common function of GH20 family glycoside hydrolases is lactose-N-biosidase activity, which acts mainly on the conversion of lactose tetrasaccharides to lactose-N-disaccharides and lactose in HMOs [9,49]. According to research on *B. infantis*, GH36 and GH42 family glycoside hydrolases typically have α-galactosidase or β-galactosidase activities, working mostly on the decreasing terminal galactose of polysaccharides and oligosaccharides [50]. Inositol 2-dehydrogenase encourages the hydrolysis of food-derived inositol in the gut and breast milk, which accelerates preterm babies’ bronchopulmonary growth and organ maturation [51]. YLGB-1496 possesses a large number of glycoside hydrolase genes, suggesting that it can utilize carbohydrates and may improve infant intestinal health if added to infant foods.

Human milk oligosaccharides (HMOs) are the third most abundant solid component in human milk. The core structures of HMOs consist of Gal, Glc, and GlcNAc, with the core frequently decorated with Fuc and N-acetylneuraminic acid (Neu5Ac) [52]. In addition to providing direct nutrition to the infant, HMOs primarily act as specific substrates for sustaining the growth of selected beneficial bacteria that aid in the development and protection of the newborn [53]. The gene clusters involved in the import and processing of HMOs were identified in *B. infantis*; the main enzymes encoded by the gene clusters are sialidase, fucosidase, N-acetyl-β-hexosaminidase, and β-galactosidase. Sialidases (EC 3.2.1.18) from *B. infantis*, which belong to GH33, act on both α-2,3 and α-2,6 linkages HMOs; it has been shown to liberate Neu5Ac from mucin glycoproteins [54,55]. Growing on HMO induces fucosidase activity; the enzyme acts on Fuc residue bound to GlcNAc [53]. Therefore, the novel gene cluster from YLGB-1496 may have been dedicated to co-regulation and participation in HMOs metabolic.

The fermentation activity of the strain is an important indicator for evaluating its fermentation performance, and its stability is essential for developing direct-vat-starter (DVS) starters [17,56]. The fermentative activity of strains is rapidly decreased by unfavorable environmental conditions, which impacts the fermentate’s stability and shelf life [57]. The periodic detection of changes in the acid-producing viability of strains during continuous subculture is an important task in strain stability research and has practical applications [31,58]. The fermentation activity of YLGB-1496 in continuous subculture was 59.3–60.70 U, indicating that the strain’s overall stability was good and that continuous subculture did not affect the strain’s ability in terms of fermentation.

SNP analysis is a crucial technique for evaluating differences in the genetic information of strains [59,60]. In comparison to more established methods like the 16S rRNA gene [61], *pheS*/*atpD* functional gene [62], ANI [47], and random amplified polymorphic DNA (RAPD) [63], SNP analysis has become an indispensable tool for studying genetic variation by detecting gene sequence and structural variation with its low-cost, time-consuming, and high-throughput characteristics [64,65]. The established standard for homogenous strains is a strain having less than 21 SNP loci during subsequent subcultures [44]. In continuous culturing for up to 1000 generations, YLGB-1496 had 20 SNP loci; this result is consistent with previous reports of LAB. There is a similarity in genomic comparisons of *Bifidobacterium bifidum* BGN4, *B. longum* JDM301, and *Lactobacillus brevis* KB 290, with the number of SNPs in consecutive cultures being less than 21 [10,31,66].

Type II secretion system represents the major pathway for exoprotein transport from the periplasm across the outer membrane in a wide variety of bacteria. Type IV pili are hair-like appendages of the proteinaceous mass that are primarily involved in adhesion, DNA uptake, motility, biofilm formation, and protein secretion and are essential for adhesion to host cells and molecules [67,68]. Typically, the Sec pathway or the twin-arginine translocation (TAT) pathway is used to deliver proteins to the cell exterior [69]. The Sec pathway recognizes unfolded protein targets and contains an N-terminal leader peptide, a hydrophobic core, and a C-terminal sequence that facilitates the binding of the Sec machinery. These proteins are either exported from the cell, or their N terminus anchors them to the membrane, depending on the peptide sequence in the C-terminal region [1,70]. LPXTG-anchored proteins are covalently bound to cell wall peptidoglycan after Sec targeting by the housekeeping sorting enzyme, sortase A (SrtA) enzyme. Sortase-dependent proteins are divided into four classes, of which class C sortases are transpeptidases and play a critical role in pilus assembly [71,72]. The genome of YLGB-1496 contains 17 Sec pathway-secreted proteins; these lipoproteins were located on the surface of the bacterial cells and could interact directly with the external environment, acting as adhesion agents.

Bifidobacteria is naturally resistant to mupirocin by expressing a specific isoleucyl-tRNA synthetase gene (*ileS*). This leads to losses in the competitive inhibition of mupirocin, which reverts the bacterium’s ability to synthesize proteins [46]. Tetracycline- and macrolide-resistant genes predominate among the resistance genes carried by bifidobacteria, e.g., *erm* (*X*), a gene encoding a ribosomal protection protein located on transposon Tn5432 in *B. animalis*, mediates its resistance to macrolides [73]; Tetracycline resistance genes such as tet (W), tet (M) and tet (O) were detected in *B. longum*, *Bifidobacterium brevis* and *B. animalis*, with tet (W) being the most prevalent. Mutations in the *rpsL* gene encoding the ribosomal subunit protein S12 in bifidobacteria can lead to high levels of resistance to streptomycin [74]. Most bifidobacteria are insensitive to aminoglycosides, which is attributed to the lack of cytochrome-mediated drug transport. No antibiotic resistance genes, including mupirocin and tetracycline, were detected in the whole genome of YLGB-1496, suggesting that it does not pose a risk of antimicrobial resistance or heteroresistance.

## 5. Conclusions

In this work, we showed that YLGB-1496 exhibits extensive carbohydrate utilization capacity and genetic stability through whole-genome sequencing based on Illumina and PacBio sequencing. An omics-based comparison of *B. infantis* revealed the presence of a wide range of glycoside hydrolase genes, with the GH13, 20, 25 family, and inositol 2-dehydrogenase being typical representatives. The 1000th-generation strain exhibited a stable fermentation activity of 60.17 U; the morphology was curved or rod-shaped, regularly arranged and well-defined, with intact cell walls that were not perforated or broken. At the 1000th generation, there were 20 SNP loci meeting the criteria of belonging to the same strain. The data obtained in this study provide insights into broad-spectrum carbohydrate utilization, genomic stability, and probiotic properties, which may enhance the industrial applications of this species.

## Figures and Tables

**Table 1:** The table shows the general genome feature comparison of seven *Bifidobacterium longum* subsp. *infantis* (*B*. *infantis*) strains. Genomic assembly statistics, along with subsystems (annotated from COG and KEGG), are displayed. The genome sequences of seven *B. infantis* strains were downloaded from the National Center for Biotechnology Information (https://www.ncbi.nlm.nih.gov/datasets/taxonomy/1682/, accessed on 1 January 2024). A comparison of genes involved in metabolism in the genomic data of the eight strains was performed using Diamond software (E-value ≤ 10^−5^).

	YLGB1496	ATCC15697	BT1	JRPT	BB02	PI007	NLS	JCM7009
General genome feature								
Size	2,758,242	2,832,748	2,578,115	2,776,348	2,757,833	2,604,585	2,598,286	2,673,222
GC content (%)	59.87	59.86	59.39	59.77	59.87	59.32	59.32	59.77
CDS No.	2442	2553	2198	2461	2441	2234	2232	2328
Number of RNAs	64	85	61	64	63	64	62	63
COG gene No.	1896	1836	1800	1847	1829	1806	1805	1846
Percent of All Genes (%)	77.64	71.92	81.89	75.05	74.93	80.84	80.87	79.3
Amino acid metabolism	117	115	117	115	114	113	113	112
Biosynthesis of other secondary metabolites	30	27	30	28	27	33	33	28
Carbohydrate metabolism	128	121	119	120	131	137	137	123
Energy metabolism	48	47	48	47	49	50	50	47
Glycan biosynthesis and metabolism	29	41	47	40	38	48	48	42
Lipid metabolism	24	25	29	27	26	28	28	29
Metabolism of cofactors and vitamins	76	73	73	73	73	75	75	75
Metabolism of other amino acids	27	27	32	27	26	27	27	28
Metabolism of terpenoids and polyketides	15	13	17	14	15	19	19	14
Nucleotide metabolism	59	60	60	62	58	59	59	63
Xenobiotics biodegradation and metabolism	11	14	12	12	13	13	13	12

**Table 2:** The table shows the growth characteristics during YLGB-1496 continuous subculture. One viability unit (U) was calculated as 1 μmol of lactic acid per 1 × 10^7^ CFU of bacterial cells fermenting 1 mL of milk under the above conditions. Tests were performed in triplicate, and the results are expressed as mean ± standard deviation (SD). Analysis of variance (ANOVA) was performed using Tukey’s range test for pairwise comparison.

Generation Time (n)	OD_600_	pH Values	Viable Bacteria Number (lg(CFU/mL))	Fermentation Activity (U)
0	2.18 ± 0.02	3.96 ± 0.01	9.45 ± 0.04	59.30 ± 0.27
200	2.16 ± 0.02	3.98 ± 0.02	9.38 ± 0.04	60.40 ± 0.38
400	2.20 ± 0.01	3.95 ± 0.01	9.41 ± 0.04	59.97 ± 0.49
600	2.20 ± 0.01	3.97 ± 0.00	9.47 ± 0.04	60.43 ± 0.35
800	2.22 ± 0.02	3.98 ± 0.01	9.37 ± 0.04	60.70 ± 0.34
1000	2.20 ± 0.01	3.96 ± 0.02	9.42 ± 0.04	60.20 ± 0.22

**Table 3:** The table shows the genomic mutation information of YLGB-1496 with different generations.

Gene Mutation Type	Generation Time (n)					
	0	200	400	600	800	1000
Single-nucleotide polymorphism (SNP)	0	14	14	18	18	20
Multiple-nucleotide polymorphism (MNP)	0	0	0	0	0	0
Insertion mutation (INS)	0	2	1	1	1	2
Deletion mutation (DEL)	0	0	0	0	0	1
Inversion mutation (INV)	0	0	0	0	0	0
Duplicate mutation (DUP)	0	0	0	0	0	0

**Table 4:** The table shows predictive analysis of lipoprotein secretion by the YLGB-1496 secretory (Sec) pathway.

Gene ID	Gene Name	Description	Transmembrane Structural Domain			COG Type
			First 60 Exp AAs No.	Total Prob of N-in	Topology	
gene0102	*urtE*	High-affinity branched-chain amino acid transport ATP-binding protein LivF	-	-	-	E
gene0139	*ilvC*	Ketol-acid reductoisomerase	-	-	-	E
gene0143	*ilvC*	ketol-acid reductoisomerase	-	-	-	E
gene0230	*ftsY*	fused signal recognition particle receptor	22.67	0.83	inside: 1–4; TMhelix: 5–27; outside: 28–420;	U
gene0352	-	FKBP-type peptidyl-prolyl cis-trans isomerase	-	-	-	-
gene0430	*ffh*	signal recognition particle subunit SRP54 [EC: 3.6.5.4]	-	-	-	U
gene0574	-	ABC transporter substrate-binding component	82.73	20.23	inside: 1–30; TMhelix: 31–48; outside: 49–62; TMhelix: 63–82; inside: 83–144; TMhelix: 145–164; outside:165–167; TMhelix: 168–190; inside: 191–286;	G
gene0631	-	ABC transporter substrate-binding component	-	-	-	G
gene0818	*bioM*	ABC transporter substrate-binding component	-	-	-	-
gene0938	-	ABC transporter substrate-binding component	-	-	-	G
gene1038	*secA*	preprotein translocase subunit SecA [EC: 7.4.2.8]	-	-	-	E; G
gene1192	secG	preprotein translocase subunit SecG	24.69	0.73	inside: 1–6; TMhelix: 7–26; outside: 27–54; TMhelix: 55–73; inside: 74–82;	E; G
gene1274	*yajC*	preprotein translocase subunit YajC	16.79	0.09	outside: 1–4; TMhelix: 5–22; inside: 23–148;	U
gene1933	-	ABC transporter substrate-binding component	-	-	-	P
gene2189	*secY*	preprotein translocase subunit SecY	20.87	1	inside: 1–16; TMhelix: 17–38; outside: 39–74; TMhelix: 75–97; inside: 98–116; TMhelix: 117–139; outside: 140–153; TMhelix: 154–176; inside: 177–187; TMhelix: 188–210; outside: 211–219; TMhelix: 220–242; inside: 243–273; TMhelix: 274–296; outside: 297–315; TMhelix: 316–338; inside: 339–373; TMhelix: 374–396; outside: 397–399; TMhelix: 400–417; inside: 418–445;	E; G
gene2304	*secE*	preprotein translocase subunit SecE	19.83	1	inside: 1–40; TMhelix: 41–63; outside: 64–75;	E; G
gene2504	*yidC*	YidC/Oxa1 family membrane protein insertase	22.58	0.41	outside: 1–46; TMhelix: 47–69; inside: 70–189; TMhelix: 190–209; outside: 210–232; TMhelix: 233–255; inside: 256–335;	U

## Data Availability

The 16s rRNA sequence of YLGB-1496 has been uploaded to the GenBank database, under the accession number OQ073725.

## Supplementary Materials

The following supporting information can be downloaded at: https://www.mdpi.com/article/10.3390/genes15040466/s1, Figure S1: Clones and morphology of YLGB-1496 in the continuous subculture; Table S1: Genes and primers used in this work; Table S2: Gnens of carbohydrate transport and metabolism of seven *B. infantis* strains; Table S3: Glycoside hydrolase specific genes of YLGB-1496; Table S4: Annotated genes related to 2′-FL utilization in eight *B. infantis* strains. Used in conjunction with Figure 5A to describe genes needed to make quantified metabolites. Included are gene name, KEGG enzyme class and pathway the gene is located in. presence in other *B. infantis* strains is noted; Table S5: Genes predicted to be involved in adhesion in YLGB-1496.
