# Supplementary figures and images for: The Genome of Bifidobacterium longum subsp. infantis YLGB-1496 Provides Insights into Its Carbohydrate Utilization and Genetic Stability
Source: Genes (Basel). 2024 Apr 8;15(4):466. doi: 10.3390/genes15040466 (PMC11154571; doi:10.3390/genes15040466)

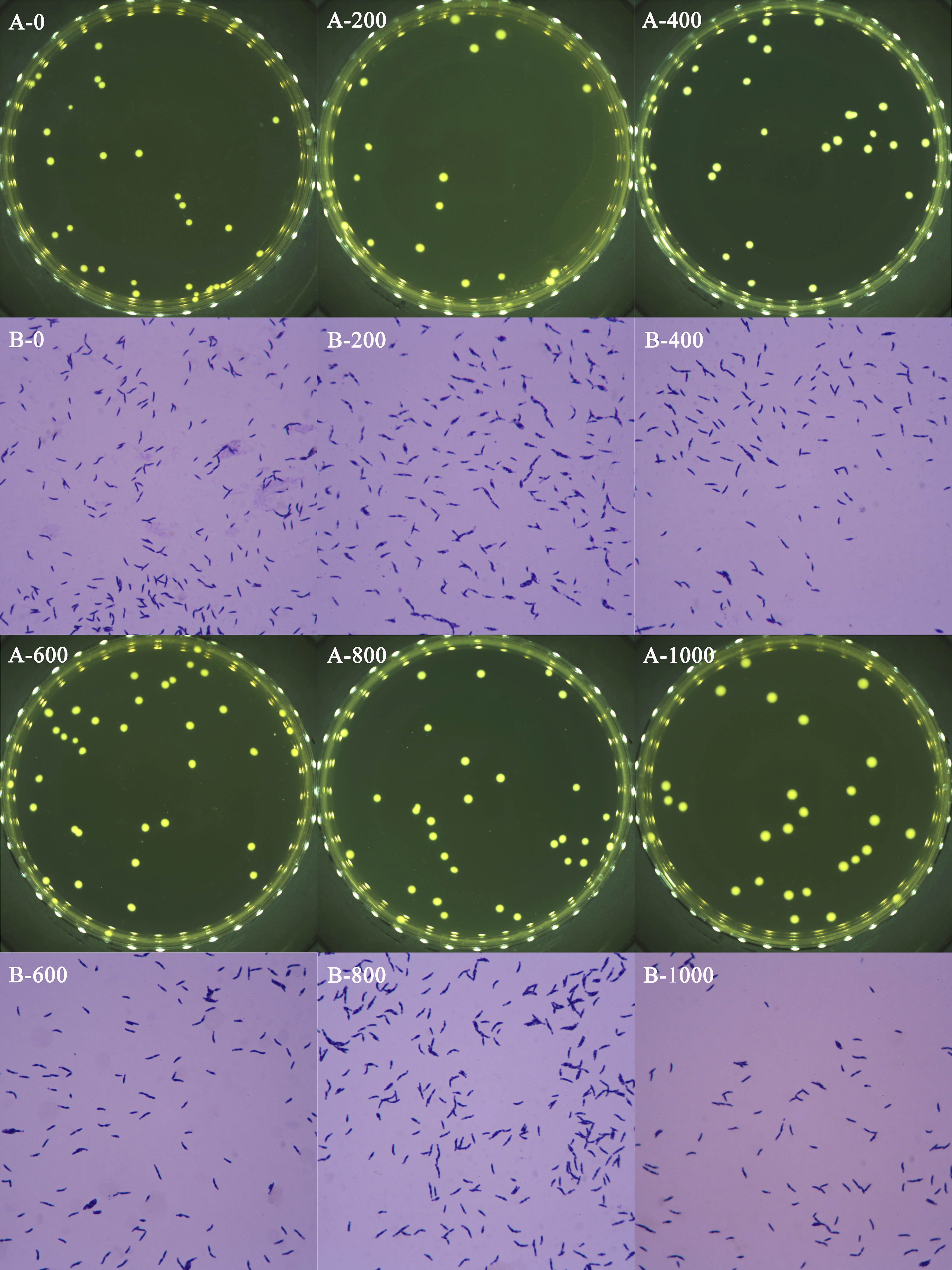

Supplement: Supplementary file 1 [file genes-15-00466-s001.zip › Figure S1.tif]
